# Comparison of Two Types of Staged Laparoscopic Orchiopexy for High Intra-Abdominal Testes in Children: A Retrospective Study From a Single Center

**DOI:** 10.3389/fped.2021.677955

**Published:** 2021-06-17

**Authors:** Jie Liu, Rui Tang, Xiao Wang, Bangzhi Sui, Zhiyuan Jin, Xudong Xu, Qinghua Zhu, Jin Chen, Honglong Ma, Guangqi Duan

**Affiliations:** Department of Pediatric Surgery, Yijishan Hospital of Wannan Medical College, Wannan Medical College, Wuhu, China

**Keywords:** laparoscopy, children, intra-abdominal testis, traction, orchiopexy

## Abstract

**Background:** To evaluate the efficacy and safety of 2nd-stage laparoscopic traction orchiopexy (Shehata technique) compared to Fowler-Stephens (F-S technique) for high intra-abdominal testes (IATs) in children.

**Patients and Methods:** We performed a retrospective review of all children (<14 years old) who underwent laparoscopic treatment of high IAT in the pediatric surgery center of Yijishan Hospital of Wannan Medical College from April 2016 to April 2020. Participants were divided into the Fowler-Stephens (F-S) group and Shehata group according to the surgical method. We collected the medical records of all children and analyzed them statistically.

**Results:** In this study, 43 patients in our center received 2nd-stage laparoscopic surgical treatment. The results showed that there were 23 high IATs in 22 patients in the F-S group and 22 IATs in 21 patients in the Shehata group. All patients completed the operation successfully. No significant difference in operation time was noted between the two groups. There was no significant difference in the testicular atrophy rate between the two groups (*P* = 0.323). The testicular retraction rate of the F-S group was greater than that of the Shehata group (*P* = 0.04).

**Conclusion:**The results of this study indicate that the application of assisted laparoscopic testicular traction technology can effectively retain the main blood supply of the testis and vas deferens with a high survival rate and clear advantages. The preliminary results show that the Shehata technique is safe, reliable and effective in the treatment of high IAT in children.

## Introduction

Cryptorchidism is a common disease in pediatric urology (1, 2). The incidence of cryptorchidism in preterm infants ranged from 9.2 to 30%, while that in full-term infants ranged from 3.4 to 5.8% (3). The main reason for this condition is that the testes do not descend from the abdominal region into the scrotal sac (4, 5). Clinically, cryptorchidism is divided into inguinal cryptorchidism and intra-abdominal testes (IATs) according to the location of testes (4, 6). Almost 20% of undescended testes are impalpable. Most of the impalpable testes are IAT (3, 7). Although B-ultrasound and magnetic resonance imaging can help to diagnose cryptorchidism, laparoscopic exploration remains an important method for the diagnosis and treatment of IAT (8).

Due to the short testicular blood vessels in the abdominal cavity, it is difficult for the testes to be lowered into the scrotum, so laparoscopic treatment of high IAT remains challenging (9). Fowler and Stephens reported for the first time in 1959 that single-stage testicular descent fixation was performed after spermatic vascular transection (10, 11). Later, the inventors improved the method. After ligating the spermatic vessels, the testes are placed in the abdominal cavity *in situ* without any treatment, and then staged surgery is performed. In addition, Shehata reported for the first time in 2008 a type of laparoscopic testicular traction and fixation for the treatment of high IAT (12). This method does not need to transect the spermatic cord blood vessels but uses the gravity of the intestine to gently and continuously provide traction to the testicular blood vessels. However, it is still debated whether Shehata surgery has more advantages than F-S surgery (13, 14).

This is a retrospective study of two surgical methods (F-S group and Shehata group) in the treatment of children with high IAT. The purpose is to evaluate the efficacy and safety of the two methods.

## Materials and Methods

From April 2016 to April 2020, we retrospectively analyzed all children (<14 years old) who underwent laparoscopic treatment of high IAT in the pediatric surgery center of Yijishan Hospital of Wannan Medical College. All the surgeries were performed by a single surgeon utilizing both techniques. In this study, the parents of all boys were objectively informed of the operation procedures, benefits and potential risks of the two types of surgery before operation. The doctors did not give any biased guidance, and the parents of each boy were free to choose the specific operation plan (F-S surgery or Shehata surgery). All data were approved by the ethics committee of Yijishan Hospital, Wannan Medical College (No. WYYJS-WZLL-13781). and the written informed consent of each boys' parents was obtained. All personal information of the children were collected in a strictly confidential and anonymous manner.

### Clinical Data

Demographic data included age at the time of surgery and side of cryptorchidism. The clinical data included the surgical method, operation time and follow-up data of the testes.

### Surgical Methods

In the F-S group, the operation procedures were performed according to the articles published by Agrawal et al. (15). In short, after entering the abdominal cavity, the lens was used to observe the location of the testis and its development, etc. There are two criteria for evaluation of testicular location: the testis, <2 cm from the ipsilateral inner ring, indicates low IAT of the abdominal cavity; on the contrary, the testis, >2 cm from the ipsilateral inner ring, indicates high IAT of the abdominal cavity. We used silk thread as a marker and put it into the abdominal cavity to measure the distance. To protect testicular collateral circulation from damage and extensive separation, the retroperitoneum was cut in the avascular area of the spermatic cord. If the testicular ischemia test is positive (we blocked the spermatic cord blood vessels with a silk thread for at least 10 min, only one knot, if there is no ischemia, it will be positive). At this time, we can make a second knot and transect the spermatic cord blood vessels. After 6 months, 2nd-stage laparoscopic testicular fixation was performed.

**Figure 1:**
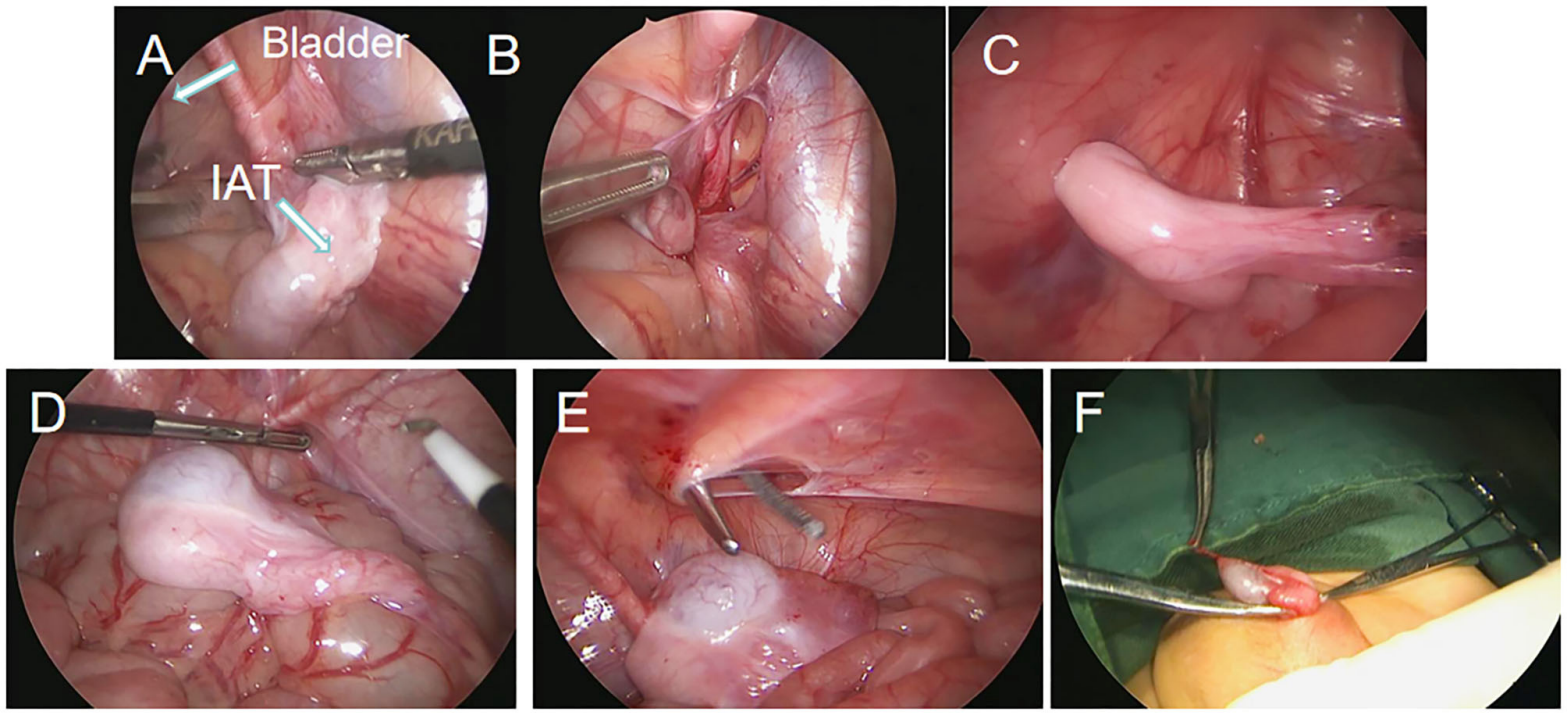
Screenshots of laparoscopic testicular traction and fixation by the Shehata technique. **(A)** Laparoscopy showed high intra-abdominal testes. **(B)** The spermatic vessels of the testes were dissociated. **(C)** The testes were fixed on the opposite abdominal wall. **(D–F)** The second stage of operation reduced the testis to scrotum and fixed the testes.

In the Shehata group, the anesthesia method, operation position and trocar position of the puncture were similar to those in the F-S group. The operation procedure was described in a paper published by Shehata (12). A detailed image of the operation is also shown in Figure 1. The testis is fixed on round needle to the fixation point one inch above and medial to the contralateral anterior superior iliac spine (ASIS) in the abdomen. A 2nd-stage laparoscopic-assisted orchiopexy was planned after 3 months.

The 2nd-stage of the two types of surgery is similar, both of which are assisted by laparoscopy, and the testicles are lowered to scrotum and fixed by incision from the scrotum on the affected side.

### Follow-Up Schedule

This study was accomplished by regular outpatient or inpatient follow-up, which included assessment of the size and location of the testes. The original case was completed by the same surgeon, and the surgeon documented immediately post-operation the development, location and size of the testis in detail. During the follow-up, the surgeon combined the clinical examination and B-ultrasound. The size and location of the testis were documented in the medical record in detail in each follow-up, and the data were entered into the follow-up data database of our center. The patients were followed up to January 2021.

### Statistical Analysis

All statistical analyses in this study were performed using the R programming language, version 3.6.0 (R Foundation), in which a *p* < 0.05 was considered statistically significant. Categorical variables were analyzed using the chi-square test, and continuous variables were analyzed using the two-sample *T*-test (independent standard *T*-test).

## Results

From April 2016 to April 2020, 46 boys with high IAT in our center received 2nd-stage laparoscopic orchiopexy surgical treatment (low IATs were excluded). There were only 3 long-looping vas patients in our study (two in F-S group and one in Shahata group). Due to the small number of patients, we excluded them in order to reduce the bias. Finally, 43 patients were included in the study. There was one patient with bilateral high IAT in each of the two groups. In total, 22 patients in the F-S group had 23 high IATs with a median age of 16 months (12–26 months), and 21 patients in the Shehata group had 22 high IATs with a median age of 17 months (12–27 months). The demographic and clinical characteristics of the two groups are shown in Table 1. No significant difference in preoperative age was noted between the two groups (*P* = 0.942). All patients were followed up for 6–48 months. The follow-up time of Shehata group and F-S group was 23.71 ± 1.487 and 22.23 ± 2.513 months respectively, and there was no significant difference between the two groups (*P* = 0.557) (Table 1).

**Table 1:** Demographic data of the Shehata and F-S technique groups.

**Variables**	**Shehata**	**F-S**	***t* (χ^2^) value**	***P*-value**
Age (Mo)	15.38 ± 2.14	15.30 ± 2.38	*t* = 0.073	0.942
Operation time, Stage 1 (min)	63.57 ± 5.78	62.75 ± 6.02	*t* = 0.347	0.731
Operation time, Stage 2 (min)	60.41 ± 5.36	60.90 ± 5.13	*t* = 0.238	0.814
PHT, 1^st^-Stage (days)	1.476 ± 0.143	1.575 ± 0.173	*t* = 0.682	0.499
PHT, 2^nd^-Stage (days)	1.483 ± 0.261	1.692 ± 0.181	*t* = 0.637	0.528
Follow-up time (Mo)	23.71 ± 1.487	22.23 ± 2.513	*t* = 0.592	0.557
Testicular atrophy	0/22	1/23 (4.3%)	χ^2^ = 0.978	0.323
Testicular retraction	0/22	4/23 (17%)	χ^2^ = 4.199	0.04

*F-S, Fowler-Stephens; Mo, month; 1^st^-Stage: first stage; 2^nd^-Stage, second stage; PHT, postoperative hospitalization time*.

**Figure 2:**
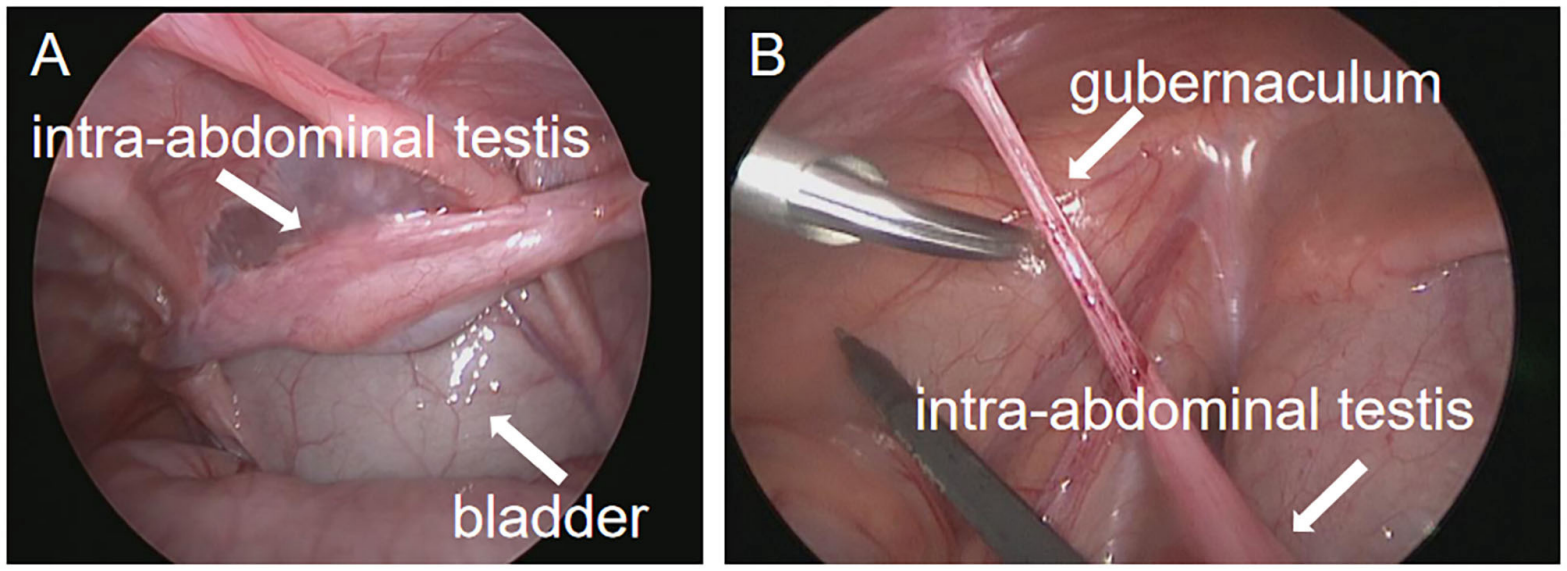
Operation images from second stage exploration in the Shehata technique. **(A)** In the second stage, the testicular spermatic vessels adhered to the bladder. **(B)** In the second stage, the gubernaculum of the testis was elongated.

All operations were carried out in all patients. In the Shehata group, one patient may have a low fixed position of the testis in the 1st-stage. As a result, in the 2nd-stage, it was found that the spermatic cord blood vessels were not in the posterior lower part of the bowel but behind the abdominal wall and adhered to the posterior lower part of the bladder (Figure 2). In another patient, the testis gubernaculum was obviously elongated in the 2nd-stage surgery (Figure 2). Fortunately, the testes of these two patients were fixed in the middle of the scrotum without tension in the 2nd-stage.

To compare the differences between the two groups, we analyzed the operation time of the 1st-stage and the 2nd-stage of the two groups. The operation time of the 1st-stage was 63.57 ± 5.78 min in the Shehata group and 62.75 ± 6.02 min in the F-S group. No significant difference was noted between the two groups (*P* = 0.942). The 2nd-stage operation time was 60.41 ± 5.36 min in the Shehata group and 60.90 ± 5.13 min in the F-S group (*P* = 0.731) (Table 1). We performed a statistical analysis on the postoperative hospitalization time (PHT) of patients after the two types of surgical methods. The results showed that the PHTs of the 1st-stage operation Shehata group and F-S group were 1.476 ± 0.143 and 1.575 ± 0.173 days, respectively, and no significant difference in PHT was noted between the two groups (*P* = 0.499). The PHTs of the 2nd-stage operation Shehata group and F-S group were 1.483 ± 0.261 and 1.692 ± 0.173 days, respectively, and there was also no significant difference in PHT between the two groups (*P* = 0.528) (Table 1). In addition, there was no testicular atrophy or retraction in the Shehata group. Although there was one case of testicular atrophy in the F-S group, there was no significant difference in the rate of testicular atrophy between the two groups (*P* = 0.323) (Table 1). In the F-S group, there were 4 cases with mild testicular retraction (the retraction distance was <1 cm during follow-up), all of which were located in the scrotum. The testicular retraction rate of the F-S group was higher than that of the Shehata group (*P* = 0.04) (Table 1).

## Discussion

Despite the fact that cryptorchidism is a common disease in pediatric surgery, the treatment of high IAT is still a great challenge for the majority of urologists (16). Many scholars attempt to solve this problem through various methods, but so far there is no consensus (17–19). F-S staged testicular fixation has been the mainstream surgical method for the treatment of high IAT for decades. However, this method has some limitations, such as two-stage waiting time is long (6 months), and spermatic cord blood vessels need to be transected (20, 21). It has been reported that laparoscopic-assisted traction testicular fixation of high IAT quickly attracted the attention and application of the majority of scholars (13, 22). Shehata improved the theory of testicular traction on the basis of previous scholars (12). Our center began to attempt to use the Shehata technique to treat high IAT in 2016. In this study, we compared the surgical results with the classic F-S technique. We have these preliminary experience.

The blood supply of the testis was effectively guaranteed by the Shehata technique because the main blood supply vessels of the testis, internal spermatic artery and vas deferens artery and vein were preserved during the operation (23). In the 1st-stage, the testis is fixed in the correct position, and the intestine pushes the spermatic cord backward and downward. With the help of chronic compression of the intestine or continuous traction of respiratory movement, spermatic cord blood vessels can be gradually extended, and testicular ischemia caused by forced pulling is avoided, which is also the greatest advantage of this technology (24). In this study, it was observed during the 2nd-stage of surgery that the spermatic cord blood vessels adhered to the bladder in one case. Fortunately, the patient's testis in the 2nd-stage of surgery smoothly fixed to the scrotum. We think that the 1st-stage of testicular fixation is very important. The testis should be fixed on the abdominal wall of the projection of the middle and upper part of the ASIS. If the fixed position is too forward, the spermatic cord will adhere to the anterior abdominal wall, which may partially affect the effect of spermatic cord lengthening. In addition, we found that if the suture was fixed on the testicular gubernaculum, the gubernaculum may be elongated and the spermatic cord blood vessels may not be fully extended in our study (Figure 2). We are worried that it may affect whether the testis can be fully lowered and fixed in the 2nd- stage operation. Thus, this procedure is not recommended. We suggest that the suture should be on the tunica albuginea of the testis in the first stage of fixation. In this study, the spermatic cord of all cases after a one-stage operation did not cause the same intestinal strangulation or compression as the abdominal cord, resulting in vascular rupture. Preliminary experience shows that this type of technique is safe, but we are also worried about whether there will be the possibility of internal hernia or intestinal obstruction with the increase in cases, which requires further exploration in future studies.

For the treatment of high IAT, laparoscopic staged F-S surgery exhibits its own special features. Specifically, after the spermatic cord blood vessels are transected, there is a long time to wait for the testicular collateral circulation (15). During the surgery, we realized that the testicular ischemia test is of great importance. However, unfortunately, an ~10% testicular atrophy rate is noted in this technique (21). The specific procedure of testicular ischemia test in this study is to block the spermatic cord blood vessels with silk thread for 10 min, pay attention to only making a knot that can be loosened again, and observe whether there are obvious changes in testicular blood supply before and after blocking. If there is no obvious ischemic change in testis after spermatic vascular occlusion, it indicates that testicular collateral blood supply is abundant and the blood supply of the gubernaculum is good, which is suitable for F-S surgery. Of course, this test is not required. According to personal habits and experience, it all depends. Our experience suggests that this test may help surgeons better to evaluate the safety of F-S surgery and reduce the risk of testicular atrophy after transection of spermatic cord blood vessels. However, the effect of testicular ischemia test on testicular atrophy has not been studied, and additional study is needed. The Shehata technique requires the spermatic cord blood vessels to be simply dissociated without being transected, which theoretically protects the testicular blood vessels, reduces the tension on the spermatic cord, and greatly reduces the risk of testicular atrophy after operation. Compared with the F-S technique, the Shehata technique also requires testicular fixation. We observed no difference in the operation time between the two techniques; that is, the Shehata technique did not significantly shorten the operation time.

The rate of testicular atrophy in the early stage of the F-S technique is very high, but the rate has been significantly reduced with the implementation of a staged surgery (11). In our study, only one case of testicular atrophy was found by B-ultrasound follow-up, which is generally consistent with the atrophy rate reported by relevant scholars (25). There was no testicular atrophy in the Shehata group potentially owed to the protection of testicular blood vessels. Some scholars reported the long-term follow-up results of testicular F-S surgery, indicating that although the testicular blood vessels were basically normal, the testicular volume was reduced (20, 26). However, long-term follow-up results of the Shehata technique are currently not available because this technique has not been employed to a not long time. However, it estimates that related research will be performed in the future. Interestingly, we found that the testicular retraction rate decreased significantly after the Shehata technique, which may be due to the effect of continuous traction. Similar to a rubber band, the retraction rate decreased after repeated stretching. In the Shehata technique, plastic tubes are used to assist traction in the initial stage. However, later, researchers thought that the mechanism of elongation might be caused by the gravity of the intestine, so they removed the plastic tube and found that it might decrease the testicular atrophy rate after improvement (24). In addition, researchers also believed that breathing with the repeated movement of abdominal muscles could also help stretch the blood vessels of the spermatic cord. On the basis of this understanding, we plan to conduct a prospective multicenter study in future research by increasing the frequency of B-ultrasound review, record the length of spermatic cord blood vessels at each time point, and use statistical methods to analyze the relationship between the length of spermatic cord blood vessels and age, the location of cryptorchidism and waiting time to further evaluate the appropriate fixation point and waiting time for staging.

In the process of testicular traction or testicular descent to the scrotum, we found that the difficulty of operation for children under 2 years old was significantly reduced. So we suggested that cryptorchidism treatment should be performed 6–12 months after birth and no more than 18 months at the latest. The distance required to lower the testicles in older boys is too long, which increases the risk and difficulty of surgery (27). We are also aware that we should strengthen the publicity of testicular physical examination, especially for the health knowledge popularization of rural children's parents. In the outpatient workup, we found that most of the children over 2 years old are from rural areas, and the parents did not realize or observe cryptorchidism. Thus, this condition was not treated at an optimal time.

At present, there is no consensus on the intraoperative confirmation criteria of high IAT. Some studies suggest that the testis in the abdominal cavity can be directly lowered and fixed within <2 cm from the inner ring (15, 28, 29). In this study, according to the latest Ain Shams classification (28, 29), we identified the high IAT from a distance of more than 2 cm from the inner ring. Earlier experience also has taught us that the IAT <2 cm away from the inner ring can be directly lowered and fixed to the scrotum. We consider our selection criteria to be appropriate. However, there are different views. Bagga et al. (30) thought that staged surgery is needed when the distance is >1 cm, Agrawal et al. (15) thought that it is necessary when the distance is <2.5 cm, and Esposito et al. (20) reported that the standard is 3 cm. We think that the selection criteria that can be used as a consensus may need further multi center, large sample and long-term follow-up study.

Regardless of the fact that Shehata technique has many advantages, it also has limitations. Abouheba et al. (13) reported that in their study, three of 34 testicles in older boys occasionally experienced testicular slippage after traction and fixation. In addition, one child with bilateral IAT was observed to have fusion of both testes concurrently during the 2nd-stage of laparoscopic surgery. Shehata et al. also reported that the success rate of traction was more than 90% in boys younger than 2 years old, and only 64% in boys older than 6 years old (24). Therefore, for the treatment of bilateral, older boys with high IAT, we need to further study and choose the appropriate surgical method.

Of course, our research still has many limitations. First, this was a single center study, so our case numbers were limited. In addition, this study is a retrospective study, and the exact operation method of all patients with high IAT in this study is informed by the surgeon to the boys' parents before operation, and their parents are free to choose. Therefore, there may be a few bias in the research results. Second, given the limited number of cases treated with Shehata technique in the preliminary study, the complications may not be completely demonstrated. Thus, further study and observation are needed. Finally, our follow-up time was not very extensive, and we have not evaluated the future fertility of the children after surgery. Thus, the long-term effects of the two types of surgery are incomparable at present.

## Conclusions

It should be noted that although there was no significant difference in the rate of testicular atrophy between Shehata surgery and F-S surgery in our study. Shehata surgery completely protected and prolonged the spermatic cord vessels and reduced the possibility of testicular atrophy. In conclusion, the application of assisted laparoscopic testicular traction technology can effectively retain the main blood supply of the testis and vas deferens with a high survival rate and obvious advantages. The preliminary results show that the Shehata technique is safe, reliable and effective in the treatment of high IAT in children.

## Data Availability Statement

The original contributions presented in the study are included in the article/supplementary material, further inquiries can be directed to the corresponding author/s.

## Ethics Statement

The studies involving human participants were reviewed and approved by the ethics committee of Yijishan hospital, Wannan Medical College. Written informed consent to participate in this study was provided by the participants' legal guardian/next of kin. Written informed consent was obtained from the individual(s), and minor(s)' legal guardian/next of kin, for the publication of any potentially identifiable images or data included in this article.

## Author Contributions

JL and GD designed the research. JL, RT and XW obtained the clinical data and analysis of the results. XX, BS and ZJ prepared the tables. QZ, JC, and HM prepared the figures. JL and GD prepared the manuscript. GD revised the manuscript. All authors have approved the final version of the manuscript.

## Conflict of Interest

The authors declare that the research was conducted in the absence of any commercial or financial relationships that could be construed as a potential conflict of interest.

## Abbreviations

IAT: intra-abdominal testes
F-S: Fowler-Stephens.
